# Interleukin-2 inducible T-cell kinase: a potential prognostic biomarker and tumor microenvironment remodeling indicator for hepatocellular carcinoma

**DOI:** 10.18632/aging.203306

**Published:** 2021-07-19

**Authors:** Binhua Pan, Modan Yang, Xuyong Wei, Wangyao Li, Kun Wang, Mengfan Yang, Di Lu, Rui Wang, Beini Cen, Xiao Xu

**Affiliations:** 1 Department of Hepatobiliary and Pancreatic Surgery, The Center for Integrated Oncology and Precision Medicine, Affiliated Hangzhou First People’s Hospital, Zhejiang University School of Medicine, Hangzhou 310006, China; 2 Zhejiang University Cancer Center, Hangzhou 310058, China; 3 Department of Hepatobiliary and Pancreatic Surgery, The First Affiliated Hospital, Zhejiang University School of Medicine, Hangzhou 310003, China; 4 NHC Key Laboratory of Combined Multi-Organ Transplantation, Hangzhou 310003, China; 5 Institute of Organ Transplantation, Zhejiang University, Hangzhou 310003, China

**Keywords:** HCC, ITK, tumor microenvironment, ESTIMATE algorithm, CIBERSORT

## Abstract

Background: The heterogeneous tumor microenvironment (TME) contributes to poor prognosis of hepatocellular carcinoma (HCC). However, determining the modulation of TME during HCC progression remains a challenge.

Methods: Herein, the stromal score and immune score of HCC samples from The Cancer Genome Atlas database were calculated using the ESTIMATE algorithm and differentially expressed genes (DEGs) were obtained. Key DEGs were identified based on a protein-protein interaction network and survival analysis. Immunohistochemistry was carried out using primary samples to evaluate key DEGs expression. The *CIBERSORT* algorithm was applied to evaluate immune components. Gene Set Enrichment Analysis (GSEA) and correlation analysis were carried out to determine the relationship between key DEGs and tumor-infiltrating immune cells (TICs).

Results: The stromal score, immune score and estimate score correlated significantly with 1-year recurrence-free survival of patients with HCC. Interleukin-2 inducible T-cell kinase (ITK) was identified as the most prognostic DEG for patients with HCC. GSEA revealed that genes in the high ITK subgroup were enriched in inflammatory-immunological terms. CIBERSORT analysis identified nine TIC subsets that correlated with ITK expression.

Conclusion: We identified ITK as a novel indicator for early post-surgery tumor recurrence and microenvironment remodeling in HCC, providing a potential therapeutic target to treat HCC.

## INTRODUCTION

Hepatocellular carcinoma (HCC) is the major form of primary liver cancer. According to authoritative statistics, liver cancer is the fourth most common cause of cancer-related death and ranks as sixth in accordance with incident cases worldwide [1]. The World Health Organization (WHO) claimed that over 1 million patients would die from liver cancer by the year 2030 [2]. HCC’s occult symptoms and highly aggressive properties mean that the majority of patients are diagnosed at an advanced stage, and the 5-year overall survival (OS) rate of patients with advanced HCC is only 25–39%, while the recurrence rate is nearly 80% [3]. Early post-operation recurrence and metastasis are the predominant cause of poor outcomes. Previous studies have reported that patients with HCC suffering early recurrence (within 1-year) after liver resection mainly had intrahepatic recurrence while patients with late recurrence commonly suffered from multi-extrahepatic metastasis, also, patients with early recurrence showed worse outcomes [4–7]. Thus, clinical management options and therapeutic efficacy for HCC are limited. Current clinical treatments for early-stage HCC include surgical resection, liver transplantation, and locoregional therapies, while systemic therapies are recommended for patients who have intermediate and advanced disease [8]. Promoted by the evolvement of multi-omics detection and analyzation techniques, reliable predictive biomarkers could guide scientific and clinical decisions that are crucial for patients with HCC [9].

The tumor microenvironment (TME) is the “soil” in which a tumor grows, as well as a severe obstacle in understanding and treating cancer. Not only cancer cells, but also immune cells, stromal cells, vascular networks, and many other components constitute the TME, representing a complex ecosystem in which cancer cells are formatted, proliferate, and progress [10]. To better determine the composition and function of the TME, several novel technologies, such as mass cytometry (CyTOF), single-cell RNA sequencing (scRNA-seq), and single-cell assay of transposase-accessible chromatin, have been adopted [11–15]. Most research has focus on tumor-infiltrating cells (TICs) especially tumor-infiltrating lymphocytes (TILs). A previous study demonstrated that the quantity of TILs in breast cancer is a robust prognostic factor for patient survival [16]. For HCC treatment, immunotherapy has recently become the new frontier of cancer treatment and the immunobiology of HCC is worthy of further exploration. Early in 2017, the landscape of infiltrating T cells was revealed by single-cell sequencing and has provided insights into immune modulation patterns [15]. Regulatory T cells (Tregs) promote tumor evasion via complicated mechanisms. The upregulation of TNF receptor superfamily member 4 (also known as OX40) expression on Tregs in the TME was reported to be associated with poor survival of patients with HCC [17]. Recent research reported that heterogeneity of exhausted T cells (Texs) in the TME is related to patient survival following resection in HCC. The authors stated that the high density of forkhead box P3 (FOXP3)^+^ Tregs in the TME correlated strongly with early tumor recurrence [18]. Results derived from previous research have increased our understanding of TME modulation of HCC and provided novel immunotherapeutic targets for further exploration.

To look deeper into the TME modulation of HCC and discover predictive biomarkers, as well as potential therapeutic targets, transcriptome-sequencing, and subsequent functional genomics analysis, are good available methods. In the current study, the ESTIMATE and CIBERSORT algorithms were used to determine the immune signatures of TICs from patients with HCC from The Cancer Genome Atlas (TCGA) database and identified interleukin-2 inducible T-cell kinase (ITK) as a predictive biomarker.

ITK belongs to the Tec family of kinases and is a crucial molecule in T cell development, differentiation, and effector function [19]. Besides its conventional participation in inflammatory activities, ITK has been reported to correlate with oncogenesis. ITK deficiency can promote severe Epstein-Barr-Virus (EBV) infection and lead to Hodgkin and non-Hodgkin lymphoma, lymphoproliferative disease, mononucleosis, and other diseases [20]. ITK expression in the TME of solid tumors is involved in the modulation of the microenvironment and is associated with prognosis. For instance, high ITK expression was found to predict better outcomes of patients with lung adenocarcinomas (LUAD) [21]. Controversially, it was reported that ITK upregulation is associated negatively with the prognosis of breast cancer [22]. As far as we know, there has been no research focusing on the correlation between ITK and the progression of HCC.

In the present study, the ESTIMATE and CIBERSORT algorithms were used to describe the immune landscape of patients with HCC. Further analysis revealed that ITK might be a potential indicator for post-operation prognosis and TME remodeling in HCC.

## RESULTS

### The stromal score, immune score, and estimate score were significantly associated with the prognosis of patients with HCC

**Figure 1:**
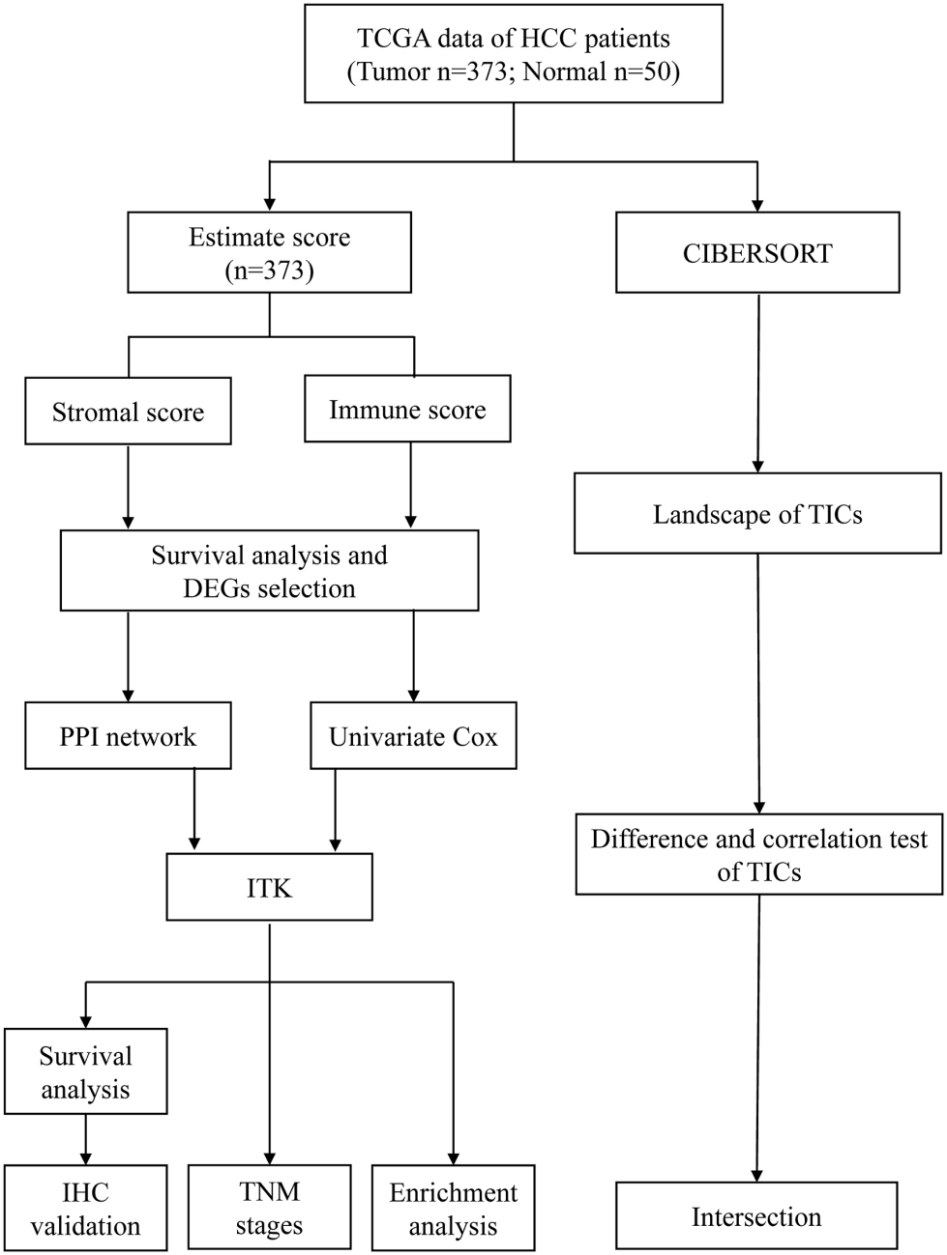
Analysis workflow of this study.

**Figure 2:**
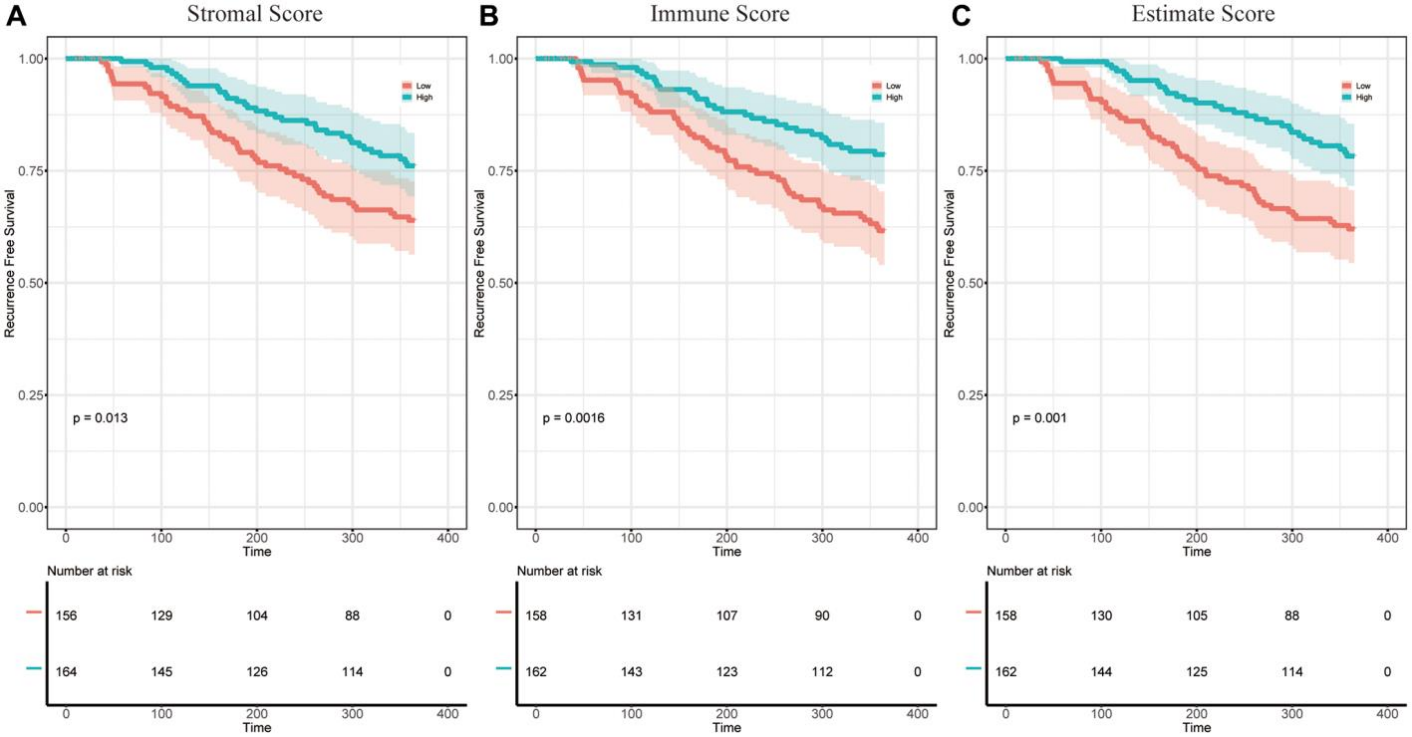
**The correlation between estimate scores and the 1-year RFS of patients with HCC.** (**A**) KM survival curves for the 1-year RFS of low/high stromal score subgroups (*p* = 0.013). (**B**) KM survival curves for the 1-year RFS of low/high immune score subgroups (*p* = 0.0016). (**C**) KM survival curves for the 1-year RFS of low/high estimate score subgroups (*p* = 0.001).

Analysis workflow of this study was shown in Figure 1. To identify the correlations among the estimated stromal and immune scores with the RFS of patients with HCC, Kaplan–Meier survival analysis was used for the stromal score, immune score, and estimate score, respectively (the clinicopathological characteristics of the patients with HCC are shown in Supplementary Table 1). According to our analysis, all three scores showed a significant correlation with the 1-year RFS rate of patients with HCC who underwent liver resection (stromal score, *p* = 0.013, Figure 2A, immune score, *p* = 0.0016, Figure 2B; estimate score *p* = 0.001, Figure 2C). We also evaluated the correlation between the scores and the long-term OS of patients with HCC; however, there was no significant difference between the low and high subgroups (Supplementary Figure 1A–1C).

### The immune score and estimate score were consistent with the clinicopathological stages of HCC

**Figure 3:**
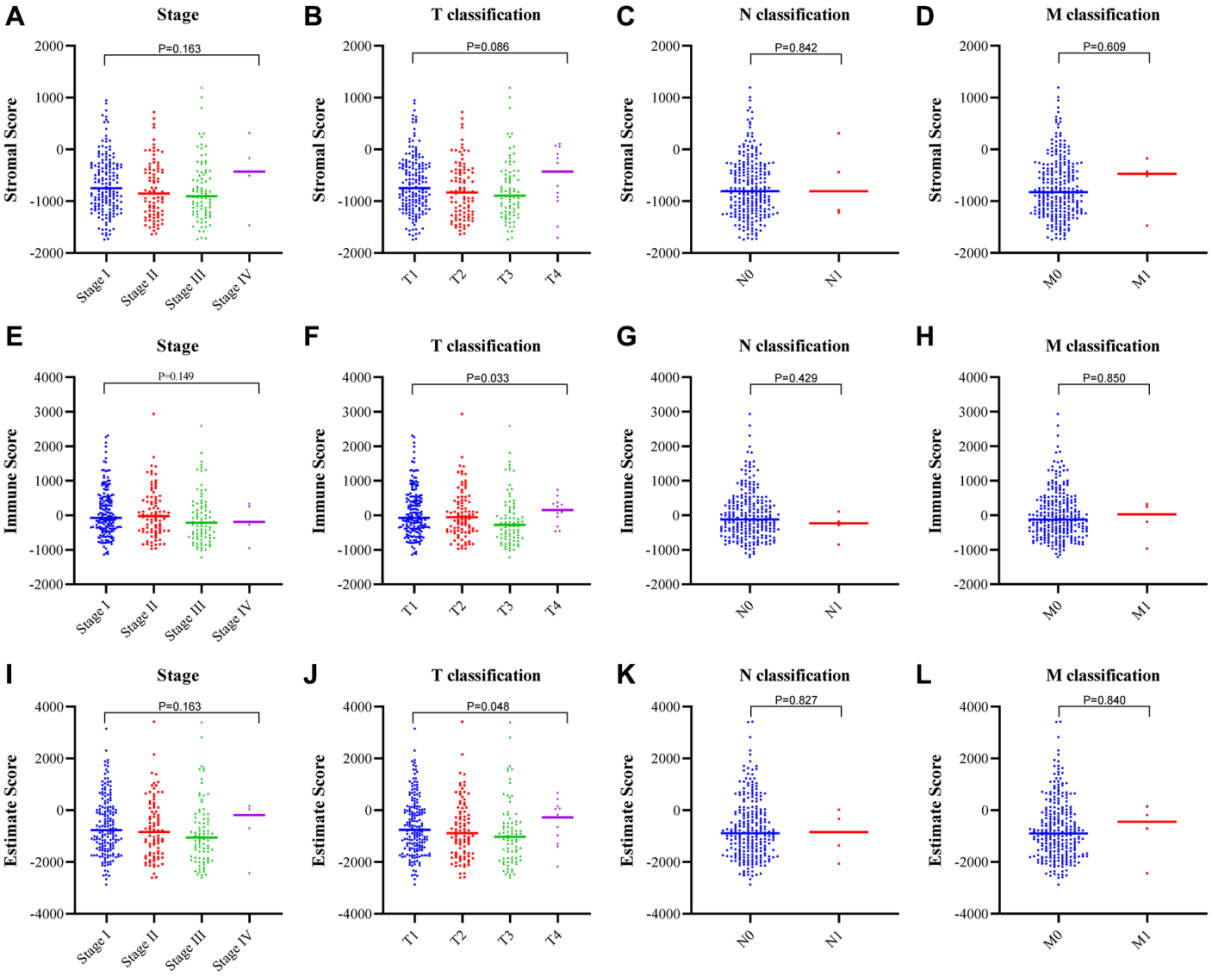
**Correlation between the estimate scores and clinicopathological staging characteristics.** (**A**–**D**) Correlation of the stromal score with the TNM stage. (**E**–**H**) Correlation of the immune score with the TNM stage. (**I**–**L**) Correlation of the estimate score with the TNM stage.

To determine the correlation between the stromal and immune scores and the clinicopathological characteristics, we downloaded the clinical data of 373 patients with HCC from the TCGA database for further analysis (Figure 3A–3L). The immune score and estimate score correlated positively with the T classification of tumor-node-metastasis (TNM) stages (Figure 3F, *p* = 0.033; Figure 3J, *p* = 0.048). While the stromal score did not correlate with any classification of HCC (Figure 3A–3D).

### Differentially expressed genes (DEGs) shared by the stromal score and immune score and their enrichment in immunity-related genes

**Figure 4:**
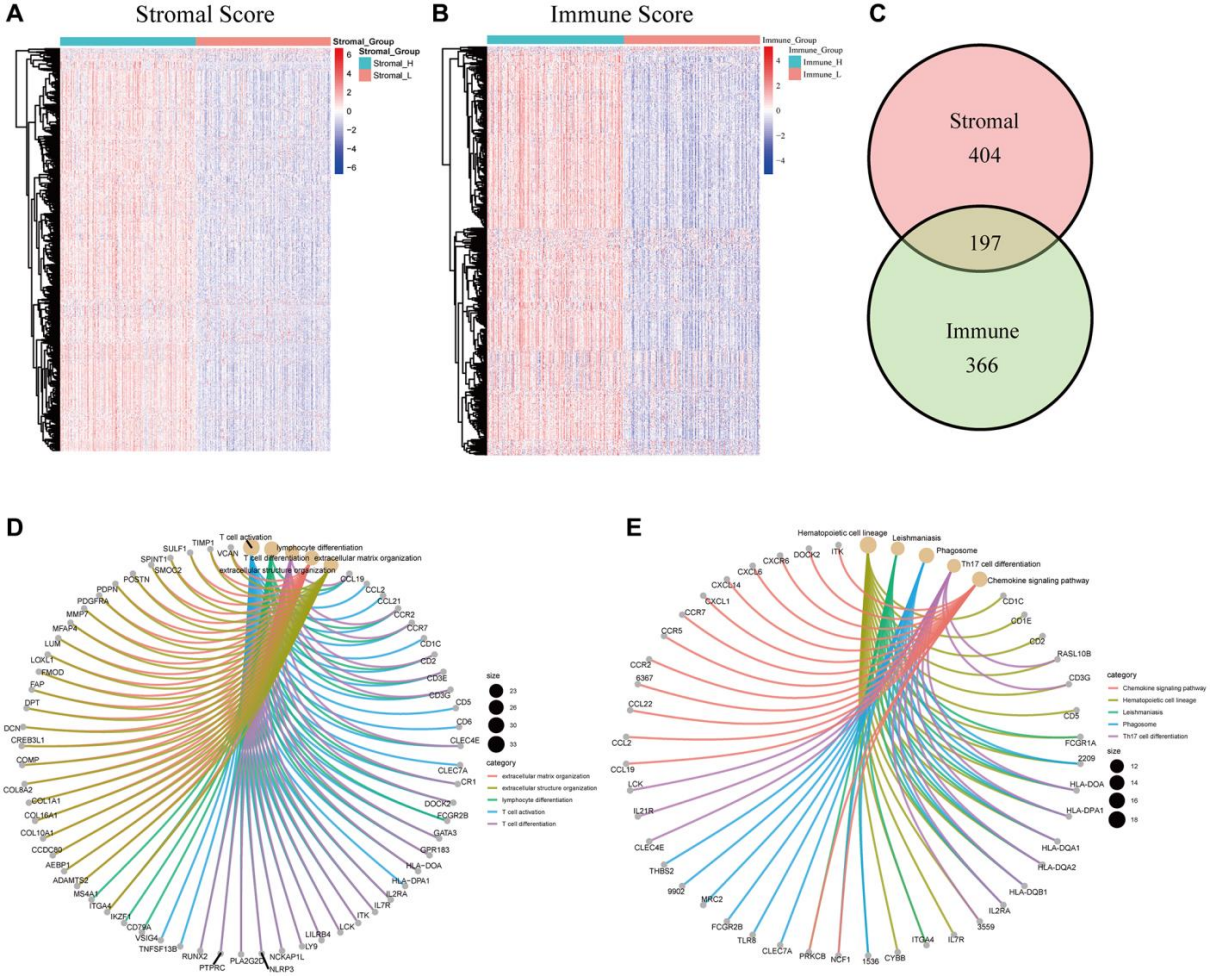
**Cluster analysis, intersection analysis, GO, and KEGG enrichment analysis of the DEGs.** (**A**) Heatmap of 601 DEGs between the high/low stromal score subgroups. (**B**) Heatmap of 563 DEGs between the high/low immune score subgroups. (**C**) Venn diagram of the DEGs commonly shared by the two groups. (**D**) GO enrichment analysis of the common DEGs. (**E**) KEGG enrichment analysis of the common DEGs.

To verify the key components in TME remodeling, we carried out comparisons between high and low stromal and immune score subgroups, respectively. 601 DEGs were obtained from the stromal score analysis, including 594 upregulated genes and 7 downregulated genes (Figure 4A). Similarly, 563 DEGs were obtained from the immune score when compared with the median, among which 557 genes were upregulated, and 6 were downregulated (Figure 4B). A Venn diagram showed that a total of 195 upregulated genes and 2 downregulated genes were shared by the high stromal score and high immune score groups (Figure 4C). These 197 DEGs were possibly the major components in TME remodeling. Gene ontology (GO) enrichment analysis showed that the DEGs were mainly enriched in terms that correlated with the immune response, such as T cell differentiation, lymphocyte differentiation, and T cell activation (Figure 4D). Likewise, the Kyoto Encyclopedia of Genes and Genomes (KEGG) and Reactome enrichment analysis showed that the DEGs were enriched in Th17 cell differentiation, the chemokine signaling pathway (Figure 4E), PD–1 signaling and immunoregulatory interactions between a Lymphoid and a non–Lymphoid cell (Supplementary Figure 2).

### A protein-protein interaction (PPI) network and univariate COX regression identified two significant factors

**Figure 5:**
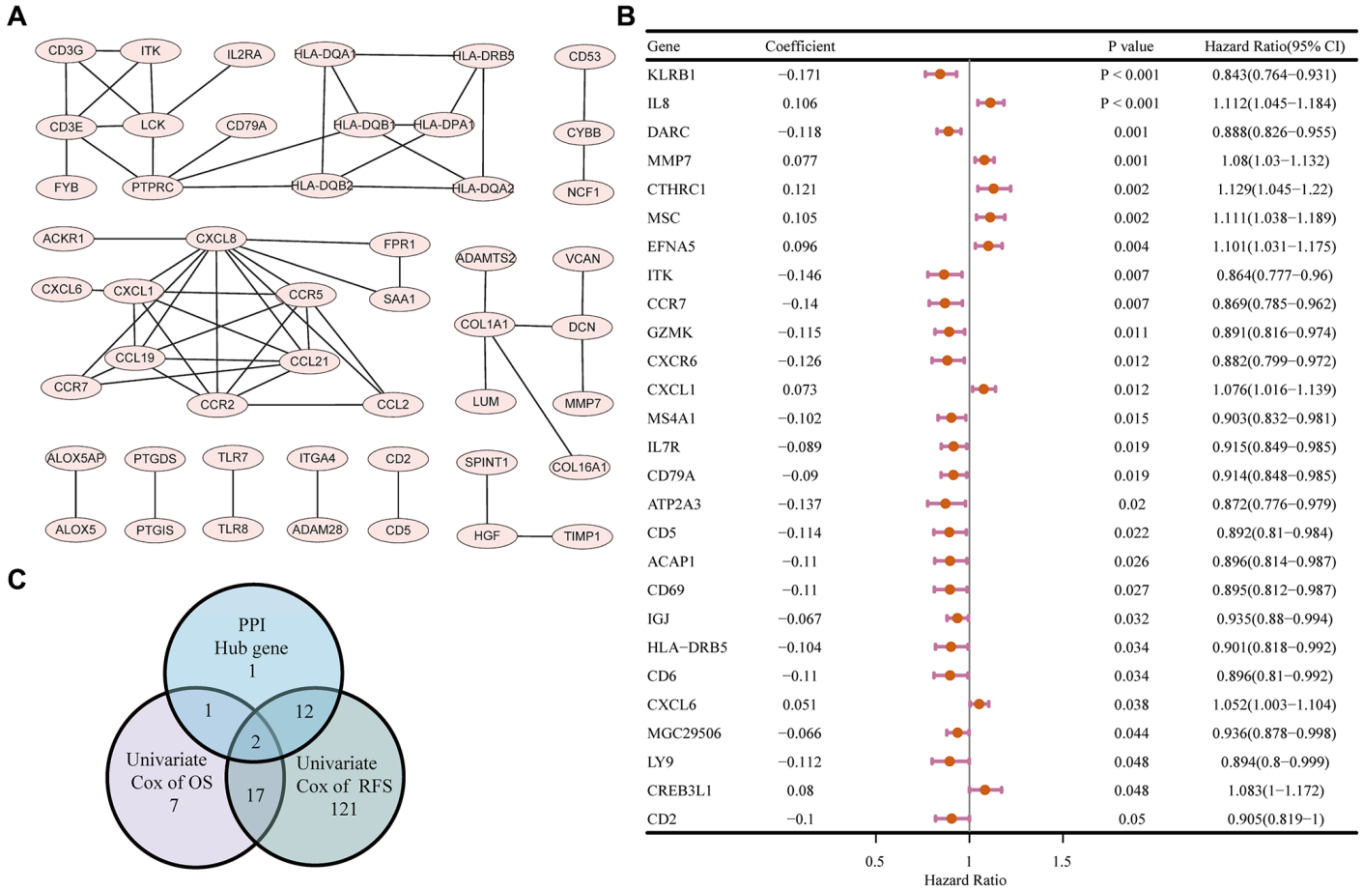
**PPI network and univariate COX regression analysis.** (**A**) PPI network of the nodes with combined score > 0.95. (**B**) Forest plot of the univariate COX regression analysis for OS. (**C**) Venn diagram of the factors commonly shared by hub genes in PPI and factors correlated with OS and RFS generated by univariate COX regression analysis.

A PPI network was then constructed based on the STRING database to explore the potential mechanisms of the DEGs in TME modulation [23]. The interactions between the protein encoded by the 197 DEGs are shown in Figure 5A. Sixteen hub genes in the PPI network were identified using MCODE, a plug-in in Cytoscape (Supplementary Table 2). Univariate COX regression analysis for the OS and RFS of patients with HCC identified 27 and 152 DEGs respectively among the 197 DEGs (Figure 5B, Supplementary Figure 3). The intersection analysis for the PPI network hub gene set, the OS-related gene set, and the RFS-related gene set was carried out next, which identified two factors, ITK and HLA Class II histocompatibility antigen DRβ5 (HLA-DRB5), as being present in the three gene sets (Figure 5C).

### ITK mRNA expression correlates with postoperative outcomes and TNM Stages for patients with HCC

**Figure 6:**
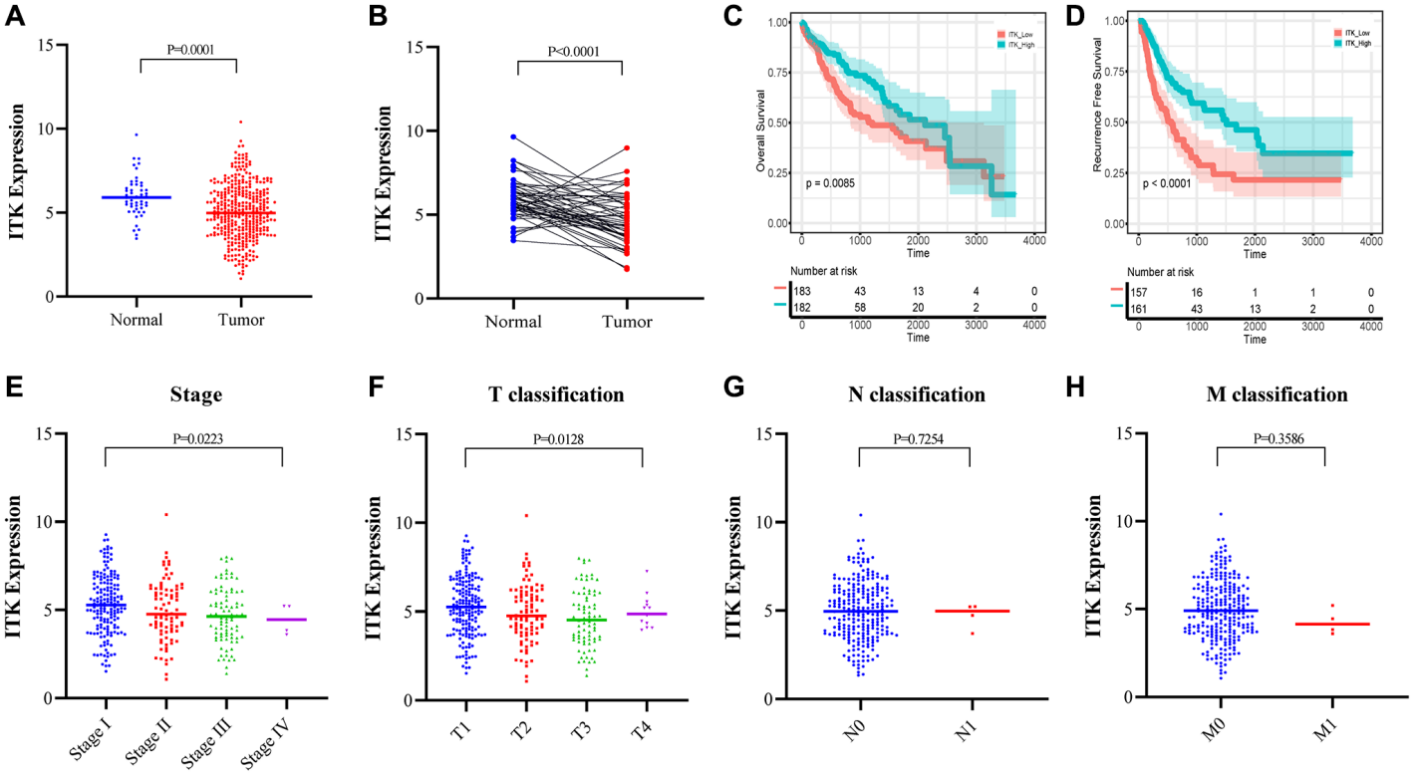
**ITK expression in HCC tumors and paracarcinoma tissues, and the correlation between ITK and survival/clinical characteristics of patients with HCC (TCGA dataset).** (**A**) ITK expression in HCC tumor tissues and paracarcinoma tissues (*p* = 0.0001). (**B**) ITK expression in paired HCC tumor tissues and paracarcinoma tissues derived from the same patient (*p* < 0.0001). (**C**) KM survival curves for the long-term OS of low/high ITK subgroups. (**D**) KM survival curves for the long-term RFS of low/high stromal score subgroups. (**E**–**H**) The correlation between ITK expression and clinicopathological stages.

The Mann–Whitney U test showed that ITK expression in HCC tumors was lower than that in normal tissues (*P* < 0.001; Figure 6A). Differential analysis of 50 paired HCC tumor and paracarcinoma tissues also revealed consistent results (*P* < 0.001; Figure 6B). All HCC samples (*n* = 365) were divided into two subgroups based on the median expression level of ITK. Kaplan–Meier survival analysis was carried out to evaluate the predictive capacity of ITK expression. According to the results, patients with HCC in the high ITK group had better 1-year, 3-year, and 5-year OS rates (88.4% vs. 77.4%, 73.5% vs. 52.8%, and 54.0% vs. 40.8%; *p* = 0.0085, Figure 6C) and RFS (80.1% vs. 59.3%, 59.4% vs. 28.8%, and 46.2% vs. 21.7%; *p* < 0.001, Figure 6D) compared with those of the ITK low group. We subsequently analyzed the correlation between ITK expression and clinical characteristics. High expression of ITK correlated with earlier clinical stages and better T classification in the TNM stage system (*P* < 0.05, Figure 6E; *P* < 0.05, Figure 6F), while there was no significant difference in ITK expression for the N and M classifications (Figure 6G and 6H). HLA-DRB5 expression showed no significant correlation with OS (*p* = 0.12, Supplementary Figure 4C) or RFS (*p* = 0.22, Supplementary Figure 4D) according to Kaplan–Meier survival analysis. HLA-DRB5 expression was not correlated with TNM staging either (Supplementary Figure 4E–4H).

### Immunohistochemistry (IHC) analysis proved that high ITK levels predict better postoperative outcomes of patients with HCC

**Figure 7:**
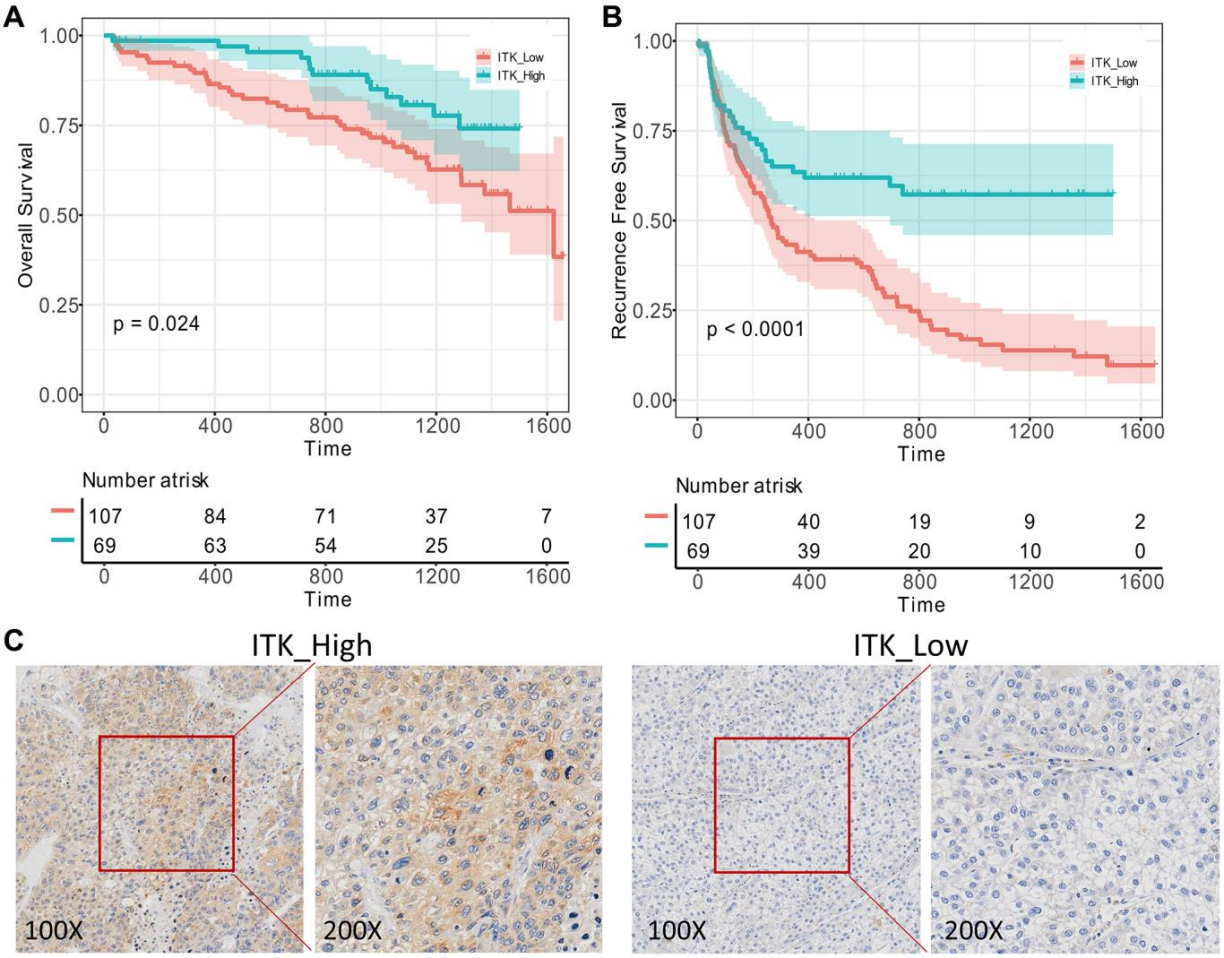
**Validation of ITK’s prognostic capacity.** 176 pairs of HCC tumor and paracarcinoma tissues were obtained from our medical center and ITK expression levels were evaluated using IHC analysis. (**A**) KM survival curves for the post-operation OS of low/high ITK subgroups (*p* = 0.024). (**B**) KM survival curves for the post-operation RFS of low/high ITK subgroups (*p* < 0.001). (**C**) IHC of ITK expression.

To further verify the prognostic capacity of ITK, we collected primary tumor samples from patients with HCC who underwent liver resection in our medical center between 2015.01.01 and 2017.12.31 (*n* = 176) and carried out IHC staining to determine ITK expression in the tissues. All the subjects were divided into a high ITK group (*n* = 69) and a low ITK group (*n* = 107) based on the IHC score, which was evaluated by two pathologists. Kaplan–Meier survival analysis showed that patients in the high ITK group had better 1-year and 3-year OS rates (97.0% vs. 89.6%, 82.9% vs. 69.0%, *p* < 0.001, Figure 7A) as well as RFS rates compared with patients in the low ITK group (63.5% vs. 41.2%, 57.3% vs. 15.4%, *p* < 0.001, Figure 7B). Representative immunohistochemical pictures of ITK expression were shown in Figure 7C.

### ITK potentially indicated TME modulation

**Figure 8:**
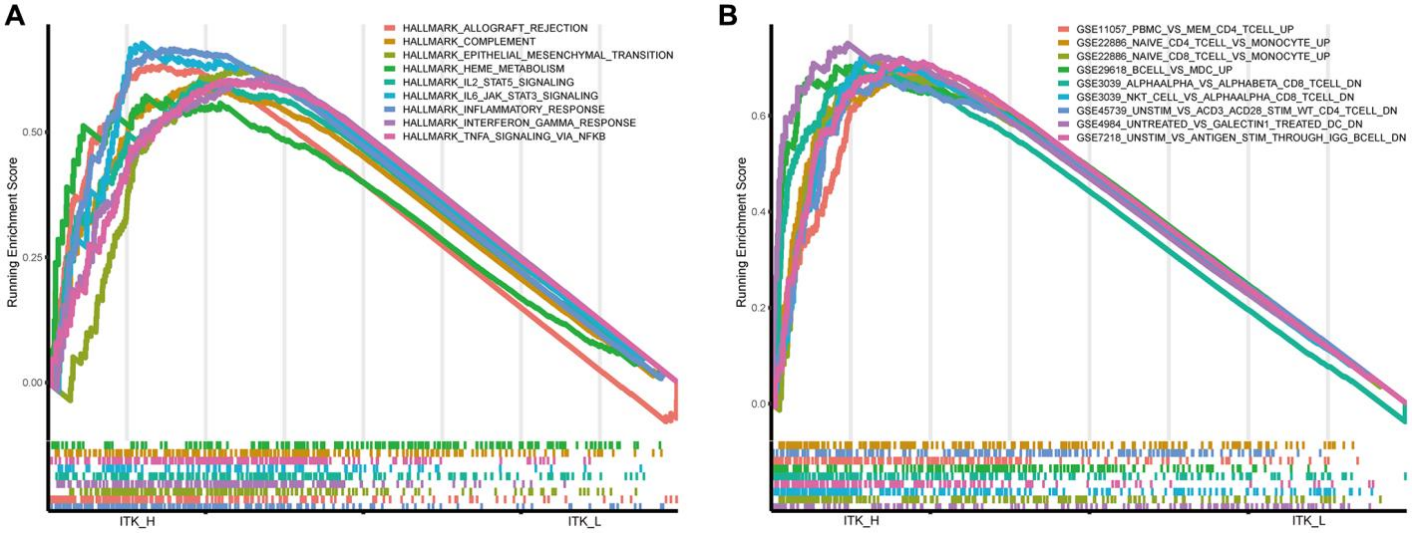
**GSEA for HCC tumor samples.** (**A**) Significantly enriched “hallmark gene sets” in the high ITK subgroup. (**B**) Significantly enriched “C7 gene sets” (the immunological gene sets) in the high ITK subgroup.

The ITK expression level was related to the prognosis and clinicopathological stages of patients with HCC; therefore, we applied Gene Set Enrichment Analysis (GSEA) analysis to determine the underlying mechanisms. As shown in Figure 8A, in the ITK high group, the genes were mainly enriched in inflammatory activities, including interleukin (IL)-2/signal transducer and activator of transcription 5 (STAT5) signaling, interferon-alpha response, and IL-6/Janus kinase (JAK)/STAT3 signaling pathways. The genes in the high ITK subgroup were enriched in multiple immunological gene sets according to the C7 collection defined by MSigDB (Figure 8B, the detailed information is shown in Supplementary Table 3).

### ITK correlated with the proportion of TIC subsets

**Figure 9:**
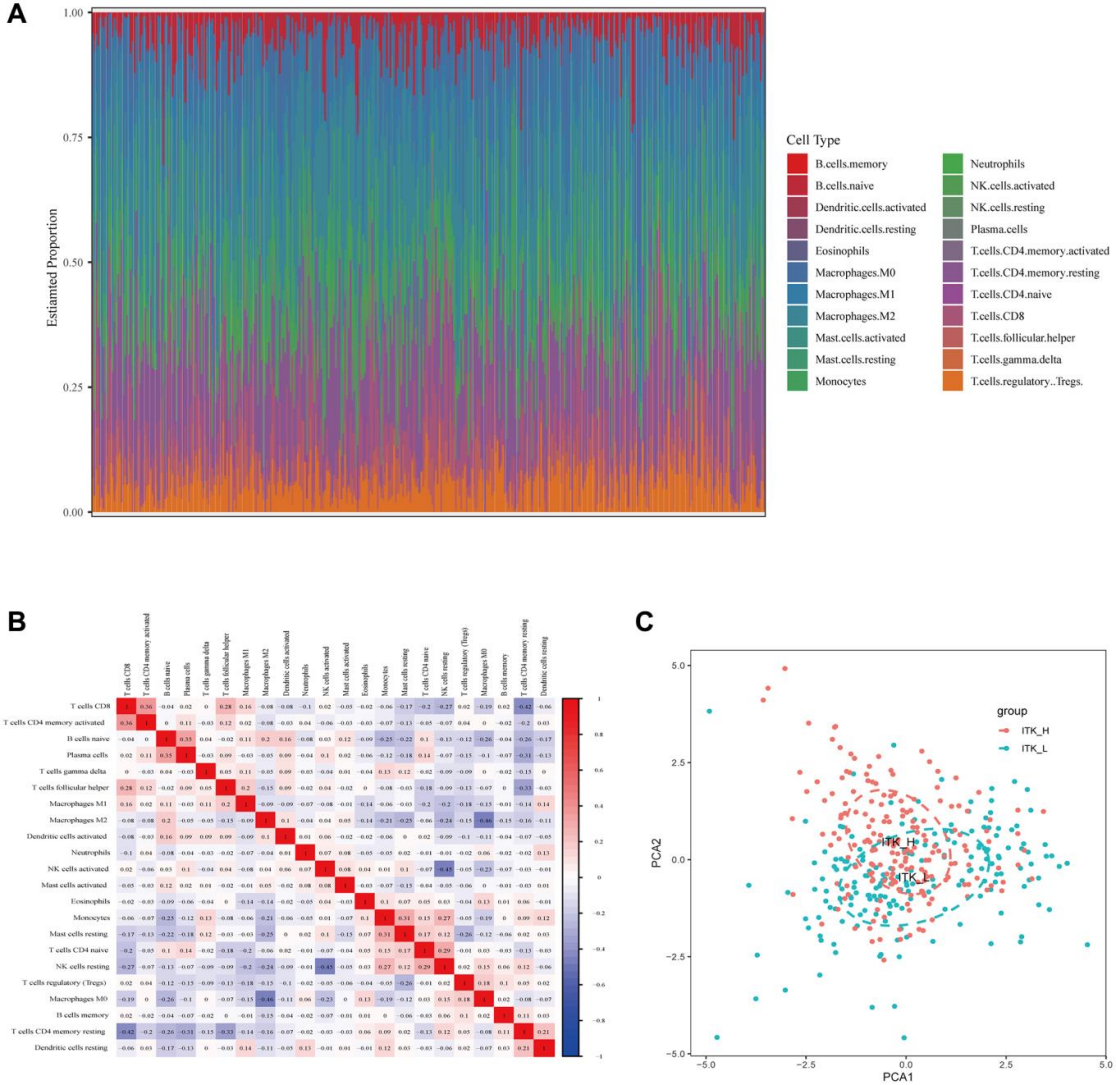
**TIC profiling of HCC tumor tissues and correlation analysis.** (**A**) The composition of 22 kinds of TICs in HCC tumor tissues is shown in a bar plot. (**B**) Heatmap showing the correlation between 22 kinds of TICs. (**C**) Principal component analysis of the HCC tumor tissues with high and low ITK expression.

**Figure 10:**
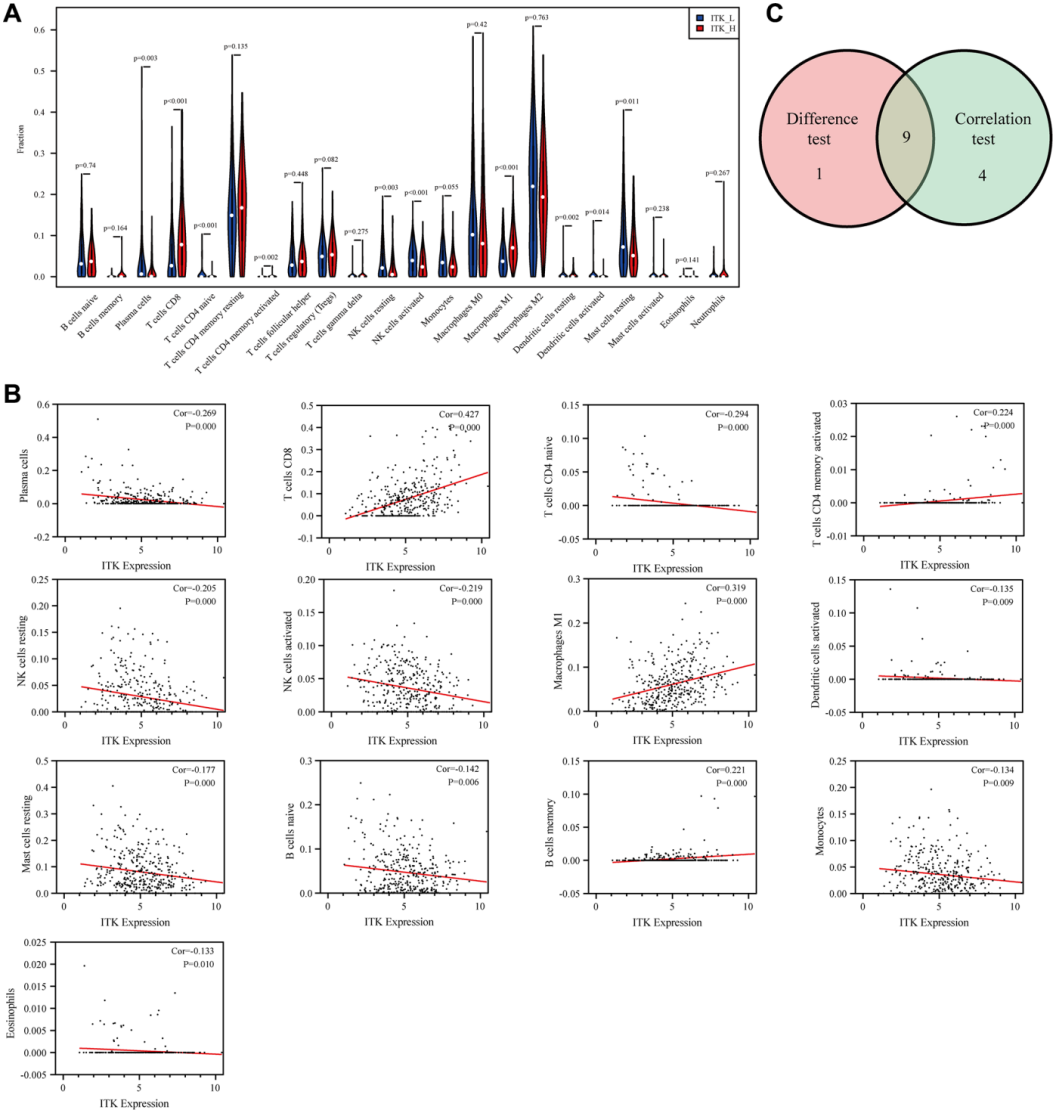
**The correlation between ITK expression and TICs proportion.** (**A**) The distributions of 22 kinds of TICs in low/high ITK subgroups are shown in a violin plot. (**B**) Scatter plots of the 13 kinds of TICs significantly correlated with ITK expression (*p* < 0.05). (**C**) Venn diagram showing nine common TICs shared, as assessed using differential analysis and correlation analysis.

We subsequently analyzed the proportion of TICs using CIBERSORT to verify the correlation of ITK expression and the immune TME. As a result, the composition of 22 kinds of immune cells in HCC tissues was calculated (Figure 9A). The correlations among the 22 immune cell subpopulations are shown in Figure 9B. The results of principal component analysis (PCA) of 373 patients with HCC are presented in Figure 9C. The difference analysis screened out 10 types of TICs (Figure 10A) and the correlation analysis identified 13 types of TICs (Figure 10B, the TICs with no correlation with ITK expression are shown in Supplementary Figure 5). Nine types of TICs overlapped in the above analyses were shown in Figure 10C and Supplementary Table 4. Among the nine TIC subsets, three of them correlated positively with ITK mRNA expression, including activated CD4^+^ memory T cells, CD8^+^ T cells, and M1 Macrophages; and six types of TICs correlated negatively with ITK expression, including plasma cells, resting natural killer (NK) cells, activated dendritic cells, activated NK cells, naïve CD4^+^ T cells, and resting mast cells. We then carried out survival analysis for the nine TIC subsets to predict postoperative OS and RFS for patients with HCC (Supplementary Figures 6–7). Results showed that CD8^+^T cells was associated with better survival, while resting NK cells, and plasma cells were associated with poor survival (*P* < 0.05). These results further supported the view that ITK is involved in immune modulation of the TME and probably exerted anti-tumor activities.

## DISCUSSION

In the present study, we identified ITK as a prognostic predictor and TME remodeling indicator for patients with HCC through comprehensive data mining based on the TCGA database. The ESTIMATE algorithm was applied to determine the predictive value of the stromal and immune scores in the TME of HCC and further screen out crucial molecules. We also depicted the landscape of the HCC TME using the CIBERSORT algorithm and revealed a correlation between the TIC composition and ITK expression. Most importantly, the results showed that ITK expression could reflect alterations in the TME of HCC and predicted the postoperative outcomes of patients with HCC.

The TME is a unique environment that emerges during tumor formation. It has been recognized that the TME, particularly its immune components, plays an important role in HCC progression and therapeutic management. Immunotherapy has been considered a novel treatment with great potential for patients with HCC. Nivolumab (Opdivo) has been approved for second-line treatment in HCC [24]. Obstructions in detecting and analyzing the comprehensive immune microenvironment of each patient in a clinical context mean that there are still obstacles in therapeutic target identification and efficacy monitoring of immunotherapy for HCC. Although we have already identified several biomarkers to predict prognosis and evaluate the efficacy of treatment for HCC, such as alpha-fetoprotein (AFP) [25], Des-Carboxy Prothrombin (DCP) [26], osteopontin (OPN) [27], vascular endothelial growth factor (VEGF) [28], and Golgi protein 73 (Gp-73) [29, 30], representative and credible biomarkers that could precisely reflect the immune modulation of the TME are highly desirable.

Inspired by the above scenario, we carried out data mining of patients with HCC based on the TCGA database from an immunological point of view. The ESTIMATE algorithm was applied to evaluate the prognostic relevance of the stromal and immune components. Our results confirmed that the immune components in TME correlated significantly with 1-year RFS of patients with HCC. We then screened out DEGs generated from the low and high stromal and immune score subgroups. The results from GO, KEGG and Reactome enrichment analysis showed that the DEGs were mainly mapped to immune-related terms, such as lymphocyte differentiation, T cell activation, and T cell differentiation. This was consistent with our initial hypothesis, as well as previous research. Subsequent intersection analysis of the PPI network and univariate COX regression analysis showed that ITK and HLA-DRB5 might be the key DEGs for HCC prognosis. HLA-DRB5 was subsequently filtered out because of its poor predictive capacity for OS of patients with HCC. Thus, we identified ITK as an immunological biomarker that performed well in evaluating the clinical stage as well as predicting the postoperative RFS and OS of patients with HCC. To further evaluate the relationship between ITK and the immune TME of HCC, functional enrichment analysis was conducted. The results of GSEA revealed that genes in the high ITK subgroup were enriched in inflammatory-immunological terms, including IL-2/STAT5 signaling, interferon-alpha response, and IL-6/JAK/STAT3 signaling pathways. Furthermore, we depicted the landscape of the HCC immune TME using the CIBERSORT algorithm, using 22 subsets of immune cells. Three kinds of TICs (CD8^+^T cells, activated CD4^+^ memory T cells, and M1 Macrophages) that correlated positively with ITK expression in HCC lesions were identified. These results were consistent with previous research findings. A recent study reported that the positive prognostic value of CD8^+^ T cells was confirmed in more than 18,700 patients across 17 solid cancer types [31]. Similar conclusions for CD4^+^ memory T cells and M1 Macrophages were published [32–36]. We also identified six TIC subpopulations, including plasma cells, resting NK cells, activated dendritic cells, activated NK cells, naïve CD4^+^ T cells, and resting mast cells, which correlated negatively with ITK expression. Among these nine TIC subsets, CD8^+^ T cells, resting NK cells, and plasma cells were related significantly to the postoperative outcomes of patients with HCC. Our results indicated that the ITK expression level could dynamically reflect antitumor immune activities.

Notably, ITK is an important member of the Tec family kinases and takes part in T cell receptor (TCR) signaling events driving processes including T cell development and Th2/Th9/Th17 responses, and mutations of ITK lead to T cell dysfunction [10, 23, 37]. Previous research found that T cell-mediated antitumor responses play an important role in natural HCC progression. For instance, tumor-associated antigen (TAA)-specific CD8^+^ T cells are significant components of HCC lesions and correlate with better RFS, indicating that the development of strategies aiming to enhance the total TAA-specific CD8^+^ T cell response would unlock their full antitumor potential [38], thus contributing to better prognosis. Likewise, the upregulation of the other two identified TICs also leads to strengthened antitumor activities in the TME of patients with HCC. Among the reduced TIC subtypes in the high ITK group, dendritic cells (DCs) are important antigen-presenting cells (APCs) and are indispensable for T cell stimulation [39]. Our results showed that ITK expression correlated negatively with DCs density, indicating the dual role played by ITK in the immune remodeling of the TME in HCC. A previous study focusing on breast cancer stated that Ibrutinib, an ITK inhibitor, could suppress tumor development and metastasis by stimulating the development of mature DCs from myeloid-derived suppressor cells (MDSCs) and might be an effective therapeutic method to treat breast cancer [40]. This is consistent with the classic therapeutic function of Ibrutinib in hematological malignancies [41]. However, controversial results were shown in solid tumors. For instance, high ITK expression is related to better prognosis in lung adenocarcinomas (LUAD) [42]. In colon cell lines and rodent models, monotherapy using the ITK inhibitor, Ibrutinib, was reported to perform poorly in tumor suppression, whereas the combination of Ibrutinib and a programmed cell death 1 ligand 1 (PD-L1) inhibitor showed significant antitumor capacity [43]. This suggested that ITK participates in the immune remodeling of the TME in HCC through complex mechanisms.

In the present study, we observed that ITK could reflect the proportion of several important immune cells within the TME of HCC, acting as a credible biomarker for the remodeling status of the antitumor composition, and thus could act as a guide for immunotherapy applications [44]. The identification of molecular targets and the determination of their functions have revealed the therapeutic value of small-molecule inhibitors. To date, 43 small-molecule inhibitors have been approved by the FDA for oncology indications [45]. According to our study, ITK presents dual function in the immune TME of HCC, and further exploration should be carried out to determine the interaction between ITK and other immune cells, as well as cancer cells. Whether an ITK inhibitor or agonist is suitable for HCC treatment in different contexts needs more robust and direct experimental evidence.

Our study also has some limitations. Although we carried out data mining based on the authoritative TCGA database, further mechanistic studies and experimental validation are required.

In conclusion, we identified ITK as a novel biomarker of RFS and TME remodeling for HCC. As a crucial molecule in T cell differentiation, ITK upregulation in HCC lesions indicates the enhanced antitumor capacity of T cells in the TME and correlated positively with better prognosis. Increasing ITK expression contributes to TME remodeling of HCC via T cell activation. We hypothesized that ITK could serve as a sensitive biomarker for the prognosis of HCC and provides a potential therapeutic target for HCC treatment in the future.

## MATERIALS AND METHODS

### Data preparation

Transcriptome RNA-seq data of 373 patients with HCC and 50 normal liver samples and the corresponding clinical data were downloaded from the TCGA database (https://portal.gdc.cancer.gov/). Our study was performed in accordance with the publication guidelines provided by the TCGA.

### Estimation of stromal and immune scores

ESTIMATE is an algorithm used to evaluate the tumor purity, which uses the gene expression of an immune signature (141 immune genes) and a stromal signature (141 stromal genes). [46] The ESTIMATE immune score and stromal score were calculated using R package estimate (v.1.0.13) [47] to analyze the infiltration levels of immune cells and stromal cells for each HCC sample, and patients with HCC were divided into high and low score groups based on the median value of immune or stromal score.

### Survival analysis

Overall survival (OS) of 365 patients with HCC and recurrence-free survival (RFS) of 320 patients with HCC were measured using the R package survival (v.3.2–3) [48] and survminer (v.0.4.8) [49]. The Kaplan–Meier method was used to plot the survival curve, and the log-rank test was used to show statistical significance, in which *p* < 0.05 was considered significant.

### Correlation analysis of the estimate score with clinical stages

The clinicopathological characteristics data of the patients with HCC were downloaded from the TCGA. SPSS (v24.0; IBM Corp., Armonk, NY, USA) was used to perform the analysis. The Mann–Whitney *U* test was used for comparison between scores and the N/M classification, and the Kruskal–Wallis rank-sum test was used to compare between scores and TNM stage/T classification, in which *p* < 0.05 was considered significant.

### Differentially expressed gene analysis

Based on the median score of the immune and stromal scores, 373 patients with HCC were subdivided into two groups (high score group and low score group), respectively. DEGs analysis was performed using R Package limma [50] to identify DEGs between the high and low score groups. DEGs with an absolute log2 fold-change >1.5 (high score vs. low score group) and an adjusted *p*-value <0.05 were considered as statistically significant.

### Functional enrichment analysis

The DEGs between the high score group and the low score group were used for GO, KEGG and Reactome enrichment analyses with the R package clusterProfiler [51] and ReactomePA [52]. Only terms with both an adjusted *p*-value and *q* value < 0.05 were considered as significantly enriched.

### PPI network construction

DEGs were imported to the STRING database to evaluate their interactive relationships, and PPI pairs with a combined score > 0.95 were then reconstructed using Cytoscape version 3.7.1 [53]. Hub genes in the PPI network were identified using MCODE, a Cytoscape plug-in.

### Gene set enrichment analysis

GSEA was performed using the R package clusterProfile with “Hallmark gene sets” and “c7 class: immunologic signature gene sets” v7.2 [54], and only gene sets with an adjusted *p*-value < 0.05 and *q*- < 0.05 were considered as significant.

### TICs profile

The CIBERSORT [55] algorithm was used to evaluate the abundance of 22 types of TICs based on the expression data from 373 tumor samples from the TCGA.

### IHC of primary samples of patients with HCC

Primary tumoral and paratumoral tissue samples of patients with HCC who underwent liver resection in our medical center between 2015.01.01 and 2017.12.31 (*n* = 176) were obtained (Ethical approval:2018–768). ITK immunohistochemistry was performed according to common protocols. The anti-ITK antibody was purchased from Abcam (Cambridge, MA, USA; No. ab32039).

## Supplementary Materials

Supplementary Figures

Supplementary Tables
