# Supplementary material for: Interleukin-2 inducible T-cell kinase: a potential prognostic biomarker and tumor microenvironment remodeling indicator for hepatocellular carcinoma
Source: Aging (Albany NY). 2021 Jul 19;13(14):18620–44. doi: 10.18632/aging.203306 (PMC8351695; doi:10.18632/aging.203306)
Supplement: Supplementary Figures [file aging-13-203306-s001.pdf]

SUPPLEMENTARY FIGURES

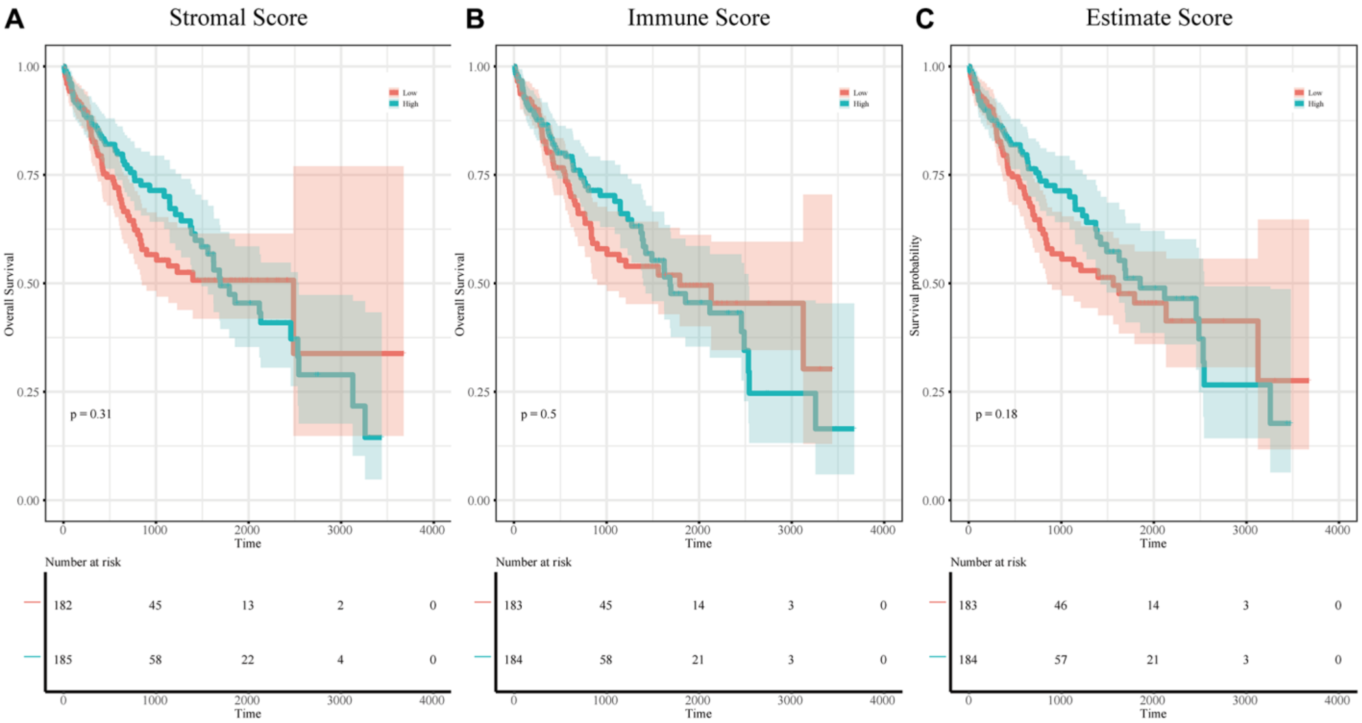

Supplementary Figure 1. The correlation between the scores and the long-term OS of patients with HCC. (**A**) KM survival curves for the long-term OS of low/high stromal score subgroups (p = 0.31). (**B**) KM survival curves for the long-term OS of low/high immune score subgroups (p = 0.5). (**C**) KM survival curves for the long-term OS of low/high estimate score subgroups (p = 0.18).

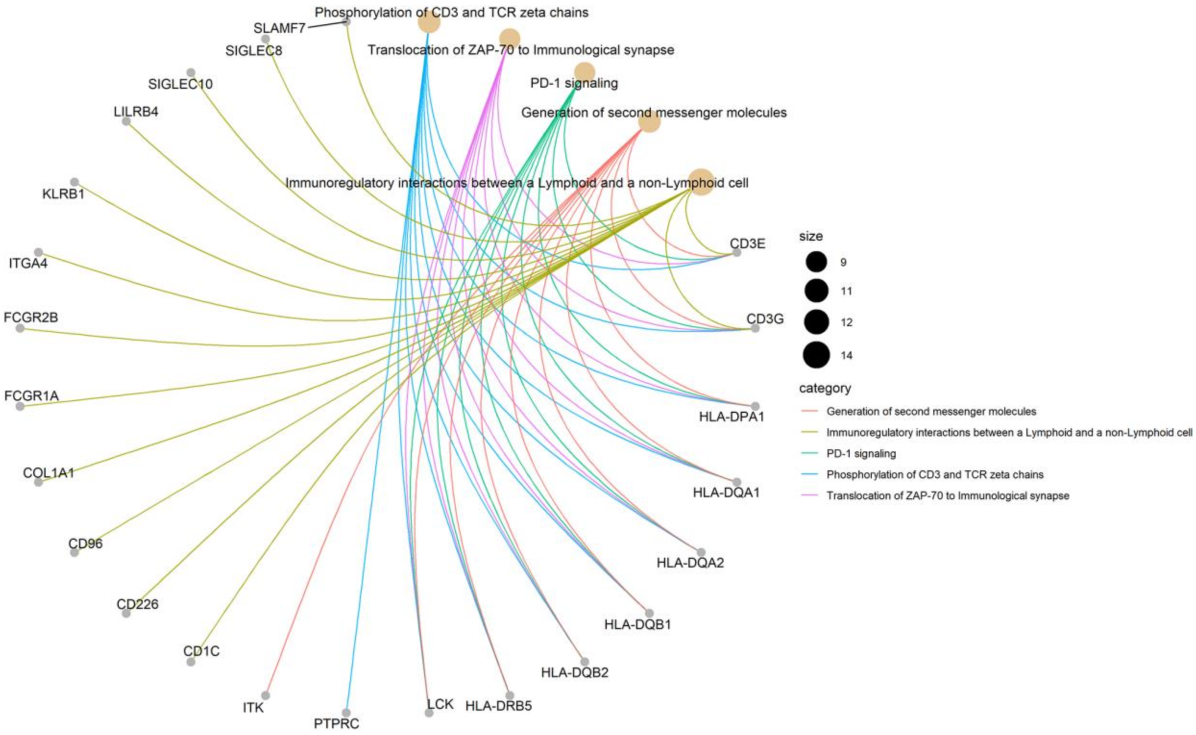

Supplementary Figure 2. Reactome enrichment analysis of the common DEGs.

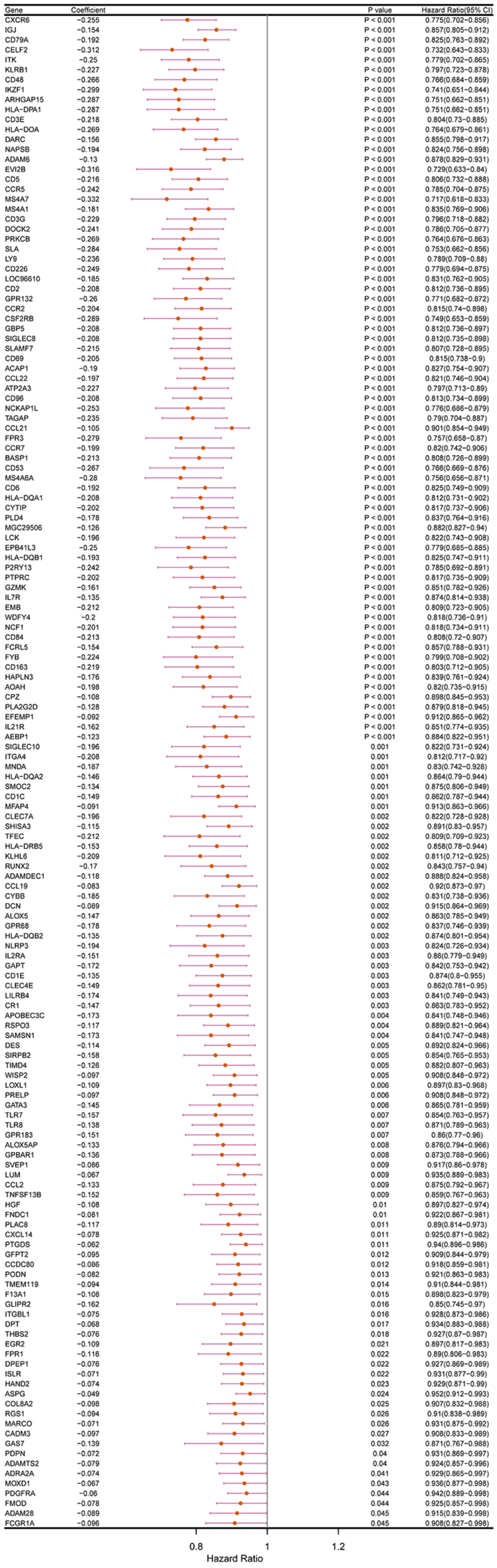

Supplementary Figure 3. Forest plot of the univariate COX regression analysis for RFS.

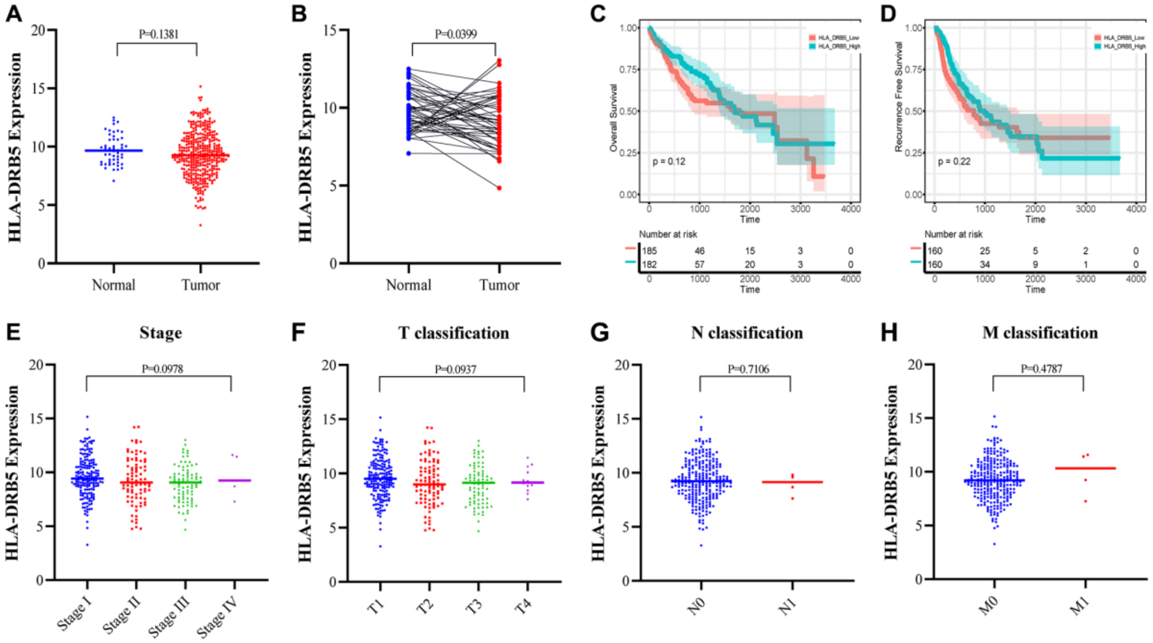

Supplementary Figure 4. HLA-DRB5 expression in HCC tumors and paracarcinoma tissues, and the correlation between HLA-DRB5 and survival/clinical characteristics of patients with HCC. (**A**) HLA-DRB5 expression in HCC tumor tissues and tumor-adjacent normal tissues (p = 0.138). (**B**) HLA-DRB5 expression in paired HCC tumor tissues and tumor-adjacent normal tissues deriving from the same patient (p = 0.040). (**C**) Overall survival of HLA-DRB5 high and low expression subgroups (p = 0.12). (**D**) Recurrence-free survival of HLA-DRB5 high and low expression subgroups (p = 0.22). (**E**–**H**) The correlation of HLA-DRB5 expression with clinicopathological staging characteristics.

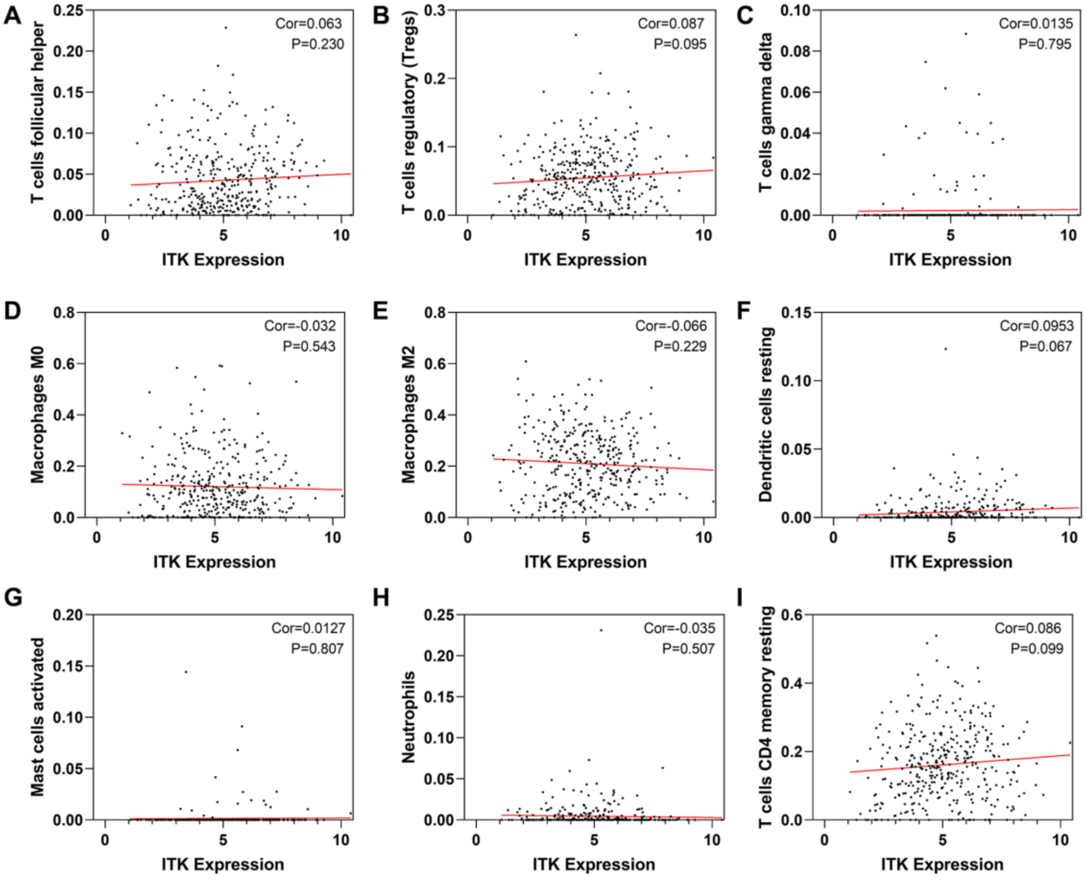

Supplementary Figure 5. Nine TICs not correlated with ITK expression.

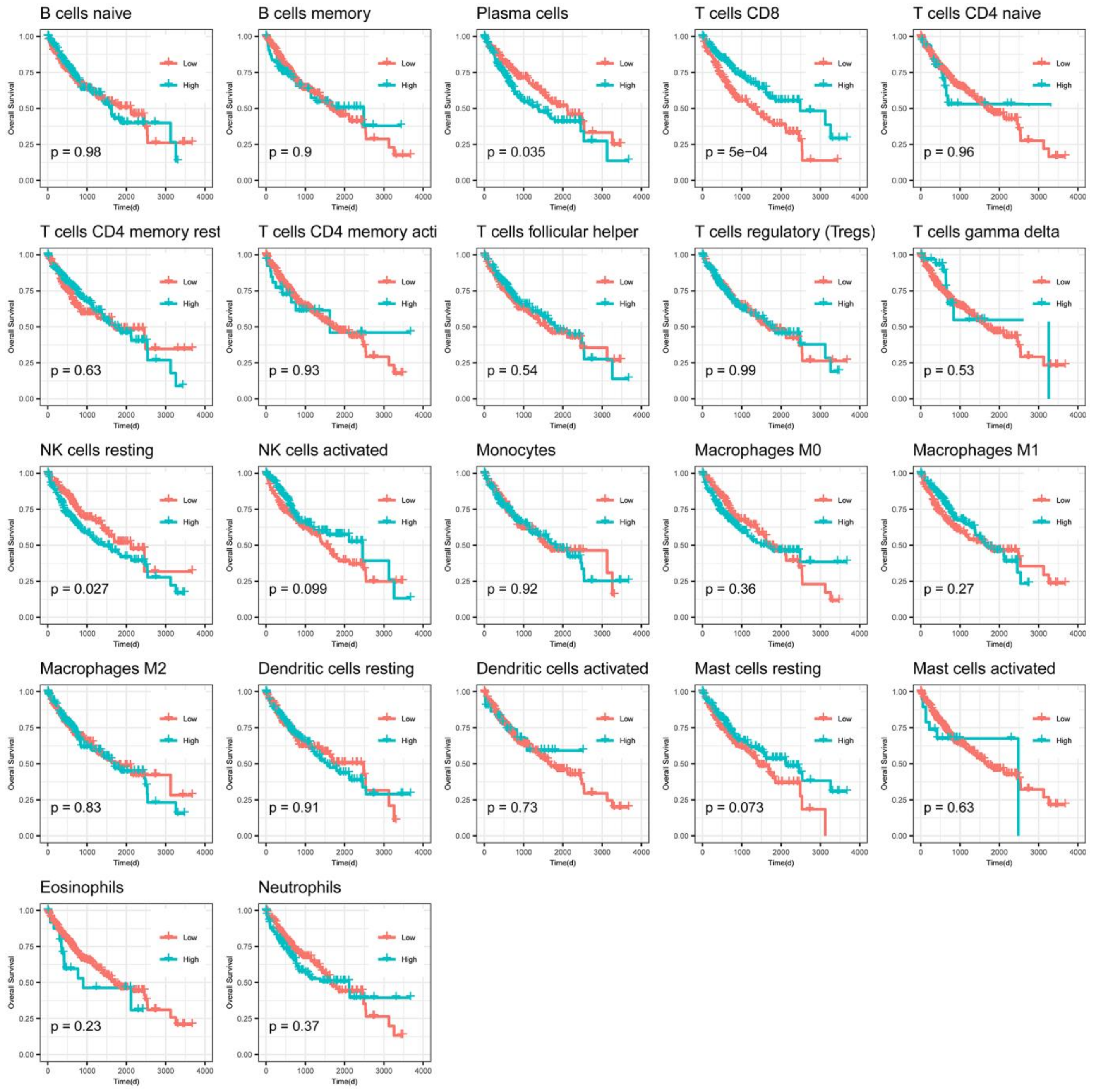

Supplementary Figure 6. KM survival curves for the long-term OS of low/high groups defined by 22 different immune cell subsets.

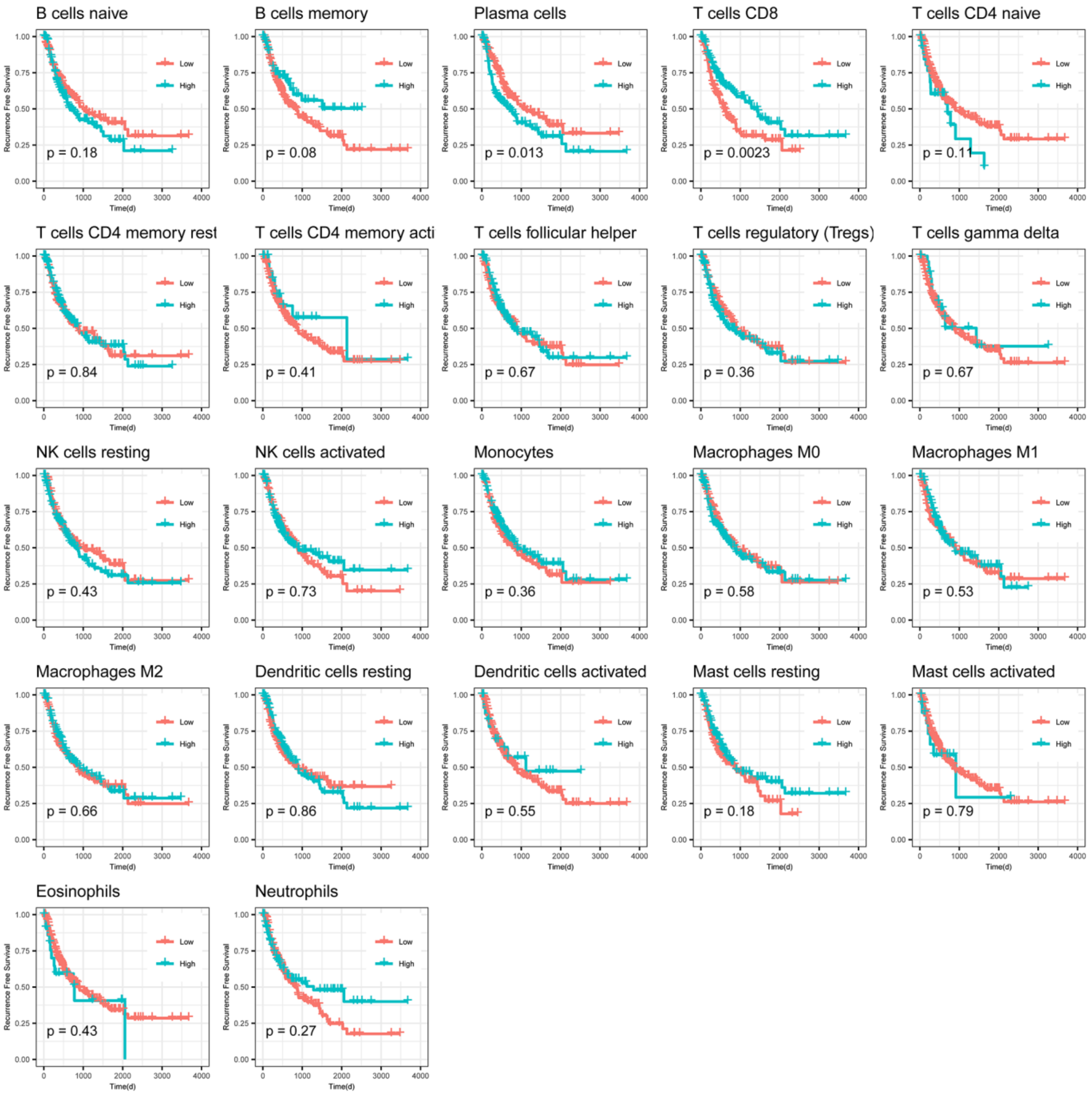

Supplementary Figure 7. KM survival curves for the RFS of low/high groups defined by 22 different immune cell subsets.
