# Supplementary material for: Interleukin-2 inducible T-cell kinase: a potential prognostic biomarker and tumor microenvironment remodeling indicator for hepatocellular carcinoma
Source: Aging (Albany NY). 2021 Jul 19;13(14):18620–44. doi: 10.18632/aging.203306 (PMC8351695; doi:10.18632/aging.203306)
Supplement: Supplementary Tables [file aging-13-203306-s002.pdf]

SUPPLEMENTARY TABLES

Supplementary Table 1. Clinicopathological characteristics statistics of LIHC patients from TCGA.

Clinical characteristics		Total	%
		373	100
Age at diagnosis (y)	young age (< = 60)	177	47.5
	old age (>60)	195	52.3
Gender	Male	252	67.6
	Female	121	32.4
Stage	I	172	46.1
	II	87	23.3
	III	85	22.8
	IV	5	1.3
T classification	T1	182	48.8
	T2	95	25.5
	T3	80	21.4
	T4	13	3.5
	TX	2	0.5
M classification	M0	267	71.6
	M1	4	1.1
	MX	102	27.3
N classification	N0	253	67.8
	N1	4	1.1
	NX	116	31.1

Supplementary Table 2. Gene list of genes identified by MCODE and univariate cox analysis.

Table A: Gene list of hub genes identified by MCODE (*n* = 16)

CCL21	CXCL8	CCR5	CCL19	CCR2	CXCL1	CD3E	LCK
ITK	CD3G	HLA-DQA2	HLA-DQB2	HLA-DRB5	HLA-DQA1	HLA-DQB1	HLA-DPA1

Table B: Genes statistically significant in univariate COX analysis of RFS (*n* = 152)

CXCR6	IGJ	CD79A	CELF2	ITK	KLRB1	CD48	IKZF1
ARHGAP15	HLA-DPA1	CD3E	HLA-DOA	DARC	NAPSB	ADAM6	EVI2B
CD5	CCR5	MS4A7	MS4A1	CD3G	DOCK2	PRKCB	SLA
LY9	CD226	LOC96610	CD2	GPR132	CCR2	CSF2RB	GBP5
SIGLEC8	SLAMF7	CD69	ACAP1	CCL22	ATP2A3	CD96	NCKAP1L
TAGAP	CCL21	FPR3	CCR7	BASP1	CD53	MS4A6A	CD6
HLA-DQA1	CYTIP	PLD4	MGC29506	LCK	EPB41L3	HLA-DQB1	P2RY13
PTPRC	GZMK	IL7R	EMB	WDFY4	NCF1	CD84	FCRL5
FYB	CD163	HAPLN3	AOAH	CPZ	PLA2G2D	EFEMP1	IL21R
AEBP1	SIGLEC10	ITGA4	MNDA	HLA-DQA2	SMOC2	CD1C	MFAP4
CLEC7A	SHISA3	TFEC	HLA-DRB5	KLHL6	RUNX2	ADAMDEC1	CCL19
CYBB	DCN	ALOX5	GPR68	HLA-DQB2	NLRP3	IL2RA	GAPT
CD1E	CLEC4E	LILRB4	CR1	APOBEC3C	RSPO3	SAMSN1	DES
SIRPB2	TIMD4	WISP2	LOXL1	PRELP	GATA3	TLR7	TLR8

GPR183	ALOX5AP	GPBAR1	SVEP1	LUM	CCL2	TNFSF13B	HGF
FNDC1	PLAC8	CXCL14	PTGDS	GFPT2	CCDC80	PODN	TMEM119
F13A1	GLIPR2	ITGBL1	DPT	THBS2	EGR2	FPR1	DPEP1
ISLR	HAND2	ASPG	COL8A2	RGS1	MARCO	CADM3	GAS7
PDPN	ADAMTS2	ADRA2A	MOXD1	PDGFRA	FMOD	ADAM28	FCGR1A

Table C: Genes statistically significant in univariate COX analysis of OS (*n* = 27)

KLRB1	IL8	DARC	MMP7	CTHRC1	MSC	EFNA5	ITK
CCR7	GZMK	CXCR6	CXCL1	MS4A1	IL7R	CD79A	ATP2A3
CD5	ACAP1	CD69	IGJ	HLA-DRB5	CD6	CXCL6	MGC29506
LY9	CREB3L1	CD2					

Supplementary Table 3. Enriched gene sets.

MSigDB collection	Description	NES	adj.*p*.value	*q*.value
h.all.v7.2.symbols.gmt				
ITK high expression	HALLMARK_INFLAMMATORY_RESPONSE	1.5482	0.0005034	0.0003921
	HALLMARK_ALLOGRAFT_REJECTION	1.4715	0.005095	0.0039687
	HALLMARK_EPITHELIAL_MESENCHYMAL_TRANSITION	1.4536	0.0055532	0.0043256
	HALLMARK_INTERFERON_GAMMA_RESPONSE	1.4195	0.0105352	0.0082064
	HALLMARK_IL2_STAT5_SIGNALING	1.3947	0.016093	0.0125356
	HALLMARK_IL6_JAK_STAT3_SIGNALING	1.4786	0.016093	0.0125356
	HALLMARK_TNFA_SIGNALING_VIA_NFKB	1.4019	0.019148	0.0149153
	HALLMARK_COMPLEMENT	1.3644	0.0281044	0.0218918
	HALLMARK_HEME_METABOLISM	1.328	0.0376875	0.0293566
c7.all.v7.2.symbols.gmt				
ITK high expression	GSE4984_UNTREATED_VS_GALECTIN1_TREATED_DC_DN	1.7377	2.37E-06	1.76E-06
	GSE22886_NAIVE_CD8_TCELL_VS_MONOCYTE_UP	1.678	9.87E-06	7.33E-06
	GSE3039_NKT_CELL_VS_ALPHAALPHA_CD8_TCELL_DN	1.6849	1.18E-05	8.75E-06
	GSE7218_UNSTIM_VS_ANTIGEN_STIM_THROUGH_IGG_BCELL_DN	1.6628	7.78E-05	5.77E-05
	GSE3039_ALPHAALPHA_VS_ALPHABETA_CD8_TCELL_DN	1.6203	0.0001806	0.0001341
	GSE29618_BCELL_VS_MDC_UP	1.6334	0.0002267	0.0001683
	GSE11057_PBMC_VS_MEM_CD4_TCELL_UP	1.6131	0.0002267	0.0001683
	GSE45739_UNSTIM_VS_ACD3_ACD28_STIM_WT_CD4_TCELL_DN	1.5972	0.0007672	0.0005696

Abbreviation: NES: normalized enrichment score; p.adjust: adjusted *p*.value; Gene sets with p.adjust less than 0.05 and *q*.value less than 0.05 were considered as statistical significance. Only several leading sets enriched in ITK high expression both in C7 were listed here due to a large number of enriched gene sets.

Supplementary Table 4. TICs co-determined by difference test and correlation test.

TICs	Pearson r	*P* (two-tailed)	Difference test (*p*-value)
Plasma cells	–0.269	<0.0001	0.003
T cells CD8	0.4274	<0.0001	<0.001
T cells CD4 naive	–0.2935	<0.0001	<0.001
T cells CD4 memory activated	0.2237	<0.0001	0.002
NK cells resting	–0.2056	<0.0001	0.003
NK cells activated	–0.2193	<0.0001	<0.001
Macrophages M1	0.3197	<0.0001	<0.001
Dendritic cells activated	–0.1351	0.0092	0.014
Mast cells resting	–0.1769	0.0006	0.011
